# Description and biological notes of the larva of *Cionus olivieri* Rosenschoeld, 1838 (Coleoptera, Curculionidae), with a comparison with other species of the tribe Cionini

**DOI:** 10.3897/zookeys.976.53930

**Published:** 2020-10-20

**Authors:** Chunyan Jiang, Roberto Caldara, Jiri Skuhrovec, Runzhi Zhang

**Affiliations:** 1 Key Laboratory of Zoological Systematics and Evolution, Institute of Zoology, Chinese Academy of Sciences, Beijing 100101, China Chinese Academy of Sciences Beijing China; 2 Center of Alpine Entomology, University of Milan, Milan, Italy University of Milan Milan Italy; 3 Group Function of Invertebrate and Plant Biodiversity in Agro-Ecosystems, Crop Research Institute, Drnovská 507, CZ-161 06 Praha 6 – Ruzyně, Czech Republic Crop Research Institute Prague Czech Republic; 4 University of Chinese Academy of Sciences, Beijing 100049, China University of Chinese Academy of Sciences Beijing China

**Keywords:** biology, Curculioninae, ecology, immature stages, *Verbascum songaricum*, weevils

## Abstract

The mature larva of *Cionus olivieri* Rosenschoeld, 1838 is described and illustrated in detail for the first time. It is compared with those known from the same genus and other genera in the tribe Cionini and with those of the hypothesized sister tribe Mecinini in the Curculioninae. The larvae of *Cionus* have three distinctive diagnostic features: the reduced number of setae on the epicranium (only two or three *des* and one or two *fs*) and on the epipharyngeal lining (only two *als*, two *ams*, and no *mes*); i.e., distinctly fewer than the most frequent number of setae in weevils, and mandibles dentate or angulate internally near the base. If considered together with *Stereonychus* Suffrian, 1854, the other genus of Cionini with larvae studied in detail, it is preliminarily suggested that mature larvae of this tribe might be characterized by six main diagnostic features: (1) labial palpi one-segmented, (2) labral rods absent, (3) pedal areas swollen to form large lobes or prolegs, (4) mandible with sharp apical teeth, (5) reduced number of *fs* on frons, only one or two *fs*, and (6) reduced number of epipharyngeal setae (two or three *als* and two or three *ams*, but no *mes*). It was noticed that *C. helleri* Reitter, 1904 from Japan, a very distinct species in the genus for some characters of the adult, also possesses distinctive characters in the larva which are uncommon among known cionines. New biological data on *C. olivieri* with the discovery of its host plant, *Verbascum songaricum* (Scrophulariaceae), in central Asia are also reported.

## Introduction

The tribe Cionini Schoenherr, 1825 in the subfamily Curculioninae Latreille, 1802 (Curculionidae) currently comprises seven genera occurring predominantly in the Palearctic region (Alonso-Zarazaga et al. 1999). The largest genus is *Cionus* Clairville, 1798, which is also represented in the Afrotropical and Oriental regions, whereas the other genera include fewer than ten species (*Cleopus* Dejean, 1821; *Nanomicrophyes* Pic, 1908; *Stereonychus* Suffrian, 1854) or are monotypic (*Cionellus* Reitter, 1904; *Patialus* Pajni, Kumar & Rose, 1991; *Stereonychidius* Morimoto, 1962). Based on the Palearctic species, Caldara and Korotyaev (2002) delimited the tribe as a monophyletic group identified by several synapomorphies in character states of the head, antennae, abdomen, and genitalia. Moreover, they also analyzed the phylogenetic relationships of the genera, based mainly on the presence of a rostral prosternal canal and tibial unci. Recently, after the revision of the Palearctic species of *Cionus* based also on a preliminary study of species of other regions, Košťál and Caldara (2019) realized that some genera seem to be paraphyletic and that most of the characters currently used to distinguish them are conflictive and need to be reassessed. On the other hand, they did not find new adult morphological characters potentially phylogenetically informative. The study of immature stages appears very important to provide additional morphological evidence.

Adults of *Cionus olivieri* Rosenschoeld, 1838 are clearly distinguished from other species of *Cionus* by the following features taken together: body size on average large (♂♂ 3.80–5.40 mm, ♀♀ 3.90–5.95 mm); rostrum in lateral view stout, almost evenly curved, approximately of the same width from base to apex, its apical part in dorsal view with parallel sides, not narrower in midlength; antennal insertion in males closer to rostrum midlength; pronotum with almost conically narrowed sides; elytra with sub-rounded sides, short (length/width ratio usually less than 1.25), with the integument concealed by densely distributed scales, without rows of erect setae-like scales; anterior onychia in males of normal length, at most as long as tarsomeres 1 to 3 combined (Košťál and Caldara 2019).

This is a widespread species in central and southern Europe and Asia. Its distribution extends from Portugal and Spain to Anatolia, Transcaucasus, Middle East, central Asia, western China (Xinjiang), Afghanistan, and Pakistan. *Cionus olivieri* does not occur in northern Europe, northern and eastern Asia, or North Africa (Alonso-Zarazaga et al. 2017, Košťál and Caldara 2019).

Host plants of Palearctic *Cionus* usually include *Verbascum*, *Scrophularia*, *Buddleja* (Scrophulariaceae) and *Limosella* (Plantaginaceae) (Hoffmann 1958; Read 1977; Räther 1989; Košťál and Caldara 2019). Larvae are ectophagous, feeding exposed on aerial parts, leaves and stems, of the plants; pupation takes place inside a cocoon built on the same host plant or in the soil (Hoffmann 1958; Smreczyński 1976; Košťál and Caldara 2019). It is known that *Cionus olivieri* lives on many *Verbascum* species: *V. thapsus* L., *V. nigrum* L., *V. sinuatum* L., *V. densiflorum* Bertol., *V. phlomoides* L., *V. longifolium* Ten. (Hoffmann 1958; Smreczyński 1976; Košťál and Caldara 2019). In Turkey, Kazakhstan, and Kyrgyzstan it was collected on a large number of *Verbascum* species (M. Košťál, pers. obs.).

The aim of the present study is to describe the larva of *C. olivieri* in detail for the first time, in order to provide further characters for the identification of this taxon, and to compare this larva with the larvae of other species of *Cionus*, Cionini, and the apparent sister tribe Mecinini Gistel, 1848.

## Materials and methods

### Sampling

Fifteen mature larvae from Kyrgyzstan (Beshtash, Talas State, 42.391391°N; 72.279285°E, 1546 m, *Verbascum songaricum*, 2-VII-2018, leg. Chunyan Jiang) and 6 mature instar larvae from Kazakhstan (Altyn Emel Conservation Area, 44.18862°N; 78.51847°E, 1577 m, *Verbascum songaricum*, 12-VIII-2018, leg. Chunyan Jiang).

### Morphological description

All described specimens were fixed in 75% ethanol and examined under a Nikon SMZ 1500 optical stereomicroscope with calibrated oculars. To prepare microscope slides, we made dissections as in May (1994): a larva was decapitated, and the head was cleared in a 10% potassium hydroxide (KOH) for three minutes by heating in a 70 °C water bath and then rinsed in distilled water. After clearing, the mouthparts were separated from the head capsule. We used nail polish as mounting medium, which contains a mixture of butyl acetate, ethyl acetate, multipolymer of adipic acid, neopentyl glycol, trimellitic acid, and acetyl tributyl citrate. All slides together with the adult specimens are deposited at the Museum of the Institute of Zoology, Chinese Academy of Sciences (**IOZ**, **CAS**).

The observations and measurements were conducted using a compound microscope with calibrated ocular lenses (Leica DM 2500). The following dimensions were measured for each larva and provided in the Description: head width and length, body length (larvae fixed in a C-shape were measured in segments), and body width in the widest place (i.e., metathorax or abdominal segments I–IV). The relative lengths of all setae can be seen in the figures. Transparent structures were stained with Chlorazol Black E for further examination.

Photos of larvae were taken with a Canon-5D camera mounted on the microscope. Images of adults were photographed with a CCD Qimaging MicroPublisher 5.0 RTV mounted on a Zeiss SteREO Discovery. V12 microscope. Images from microscopic slides were taken with a Nikon CoolPix 5400. Photographs in the field were taken with Canon G15 camera.

Drawings were made from the original photographs using the software Adobe Illustrator CS6. The numbers of setae in bilateral structures are given for one side only.

We used the terms and abbreviations for the setae of the mature larvae found in May (1994) and Marvaldi (1999).

## Results

### Cionus olivieri

Taxon classificationAnimaliaColeopteraCurculionidae

Rosenschoeld, 1838

FF429B55-02FC-52DC-8FBB-A8C9F246892B

Cionus olivieri Rosenschoeld, 1838: 725. Hoffmann 1958: 1222. Smreczyński 1976: 58. Košťál and Caldara 2019: 68.

#### Description of mature larva.

Figures 1–12. *Measurements* (in mm). Body length: 9.00–9.75 (*N* = 18). Body width: 2.50–3.00 (*N* = 18, abdominal segment III–V). Head width (in front view): 0.68–0.74 (*N* = 10); length: 0.70–0.78 (*N* = 10).

**Figures 1–4.**
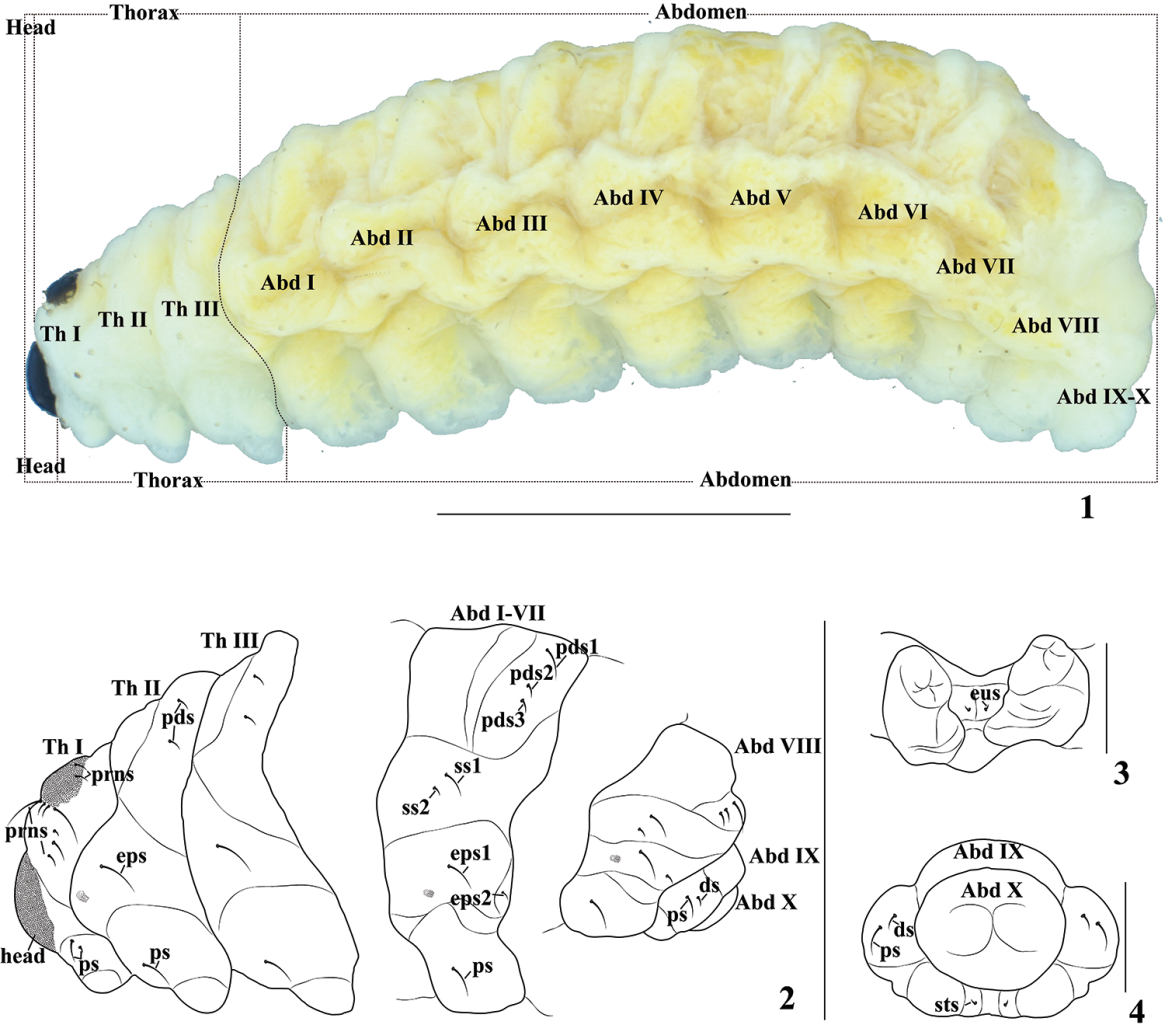
*Cionus olivieri*, mature larva: **1** habitus, lateral view **2** thoracic segments, abdominal segment I, and abdominal segments VIII–X, lateral view **3** pedal area, ventral view **4** anus (ThI–III, numbers of thoracic segments; AbdI–X, numbers of abdominal segment. Setae: *ds* dorsal s., *eps* epipleural s., *eus* eusternal s., *pds* postdorsal s., *prns* pronotal s., *ps* pleural s., *ss* spiracular s., *sts* sternal s.). Scale bars: 2 mm (**1, 2**), 1 mm (**3, 4**).

*General.* Body subcylindrical, C-shaped, weakly curved, extremely soft, declivous and attenuate posteriorly (Figs 1, 13).

*Coloration.* Head dark brown, very strongly sclerotized (Figs 1, 13). All thoracic and abdominal segments yellow, pronotum partly pigmented and sclerotized (Figs 1, 13).

*Vestiture.* Thoracic and abdominal segments with some minute and relatively long setae, cuticle minutely spiculate, pleural lobes distinct.

**Figure 5.**
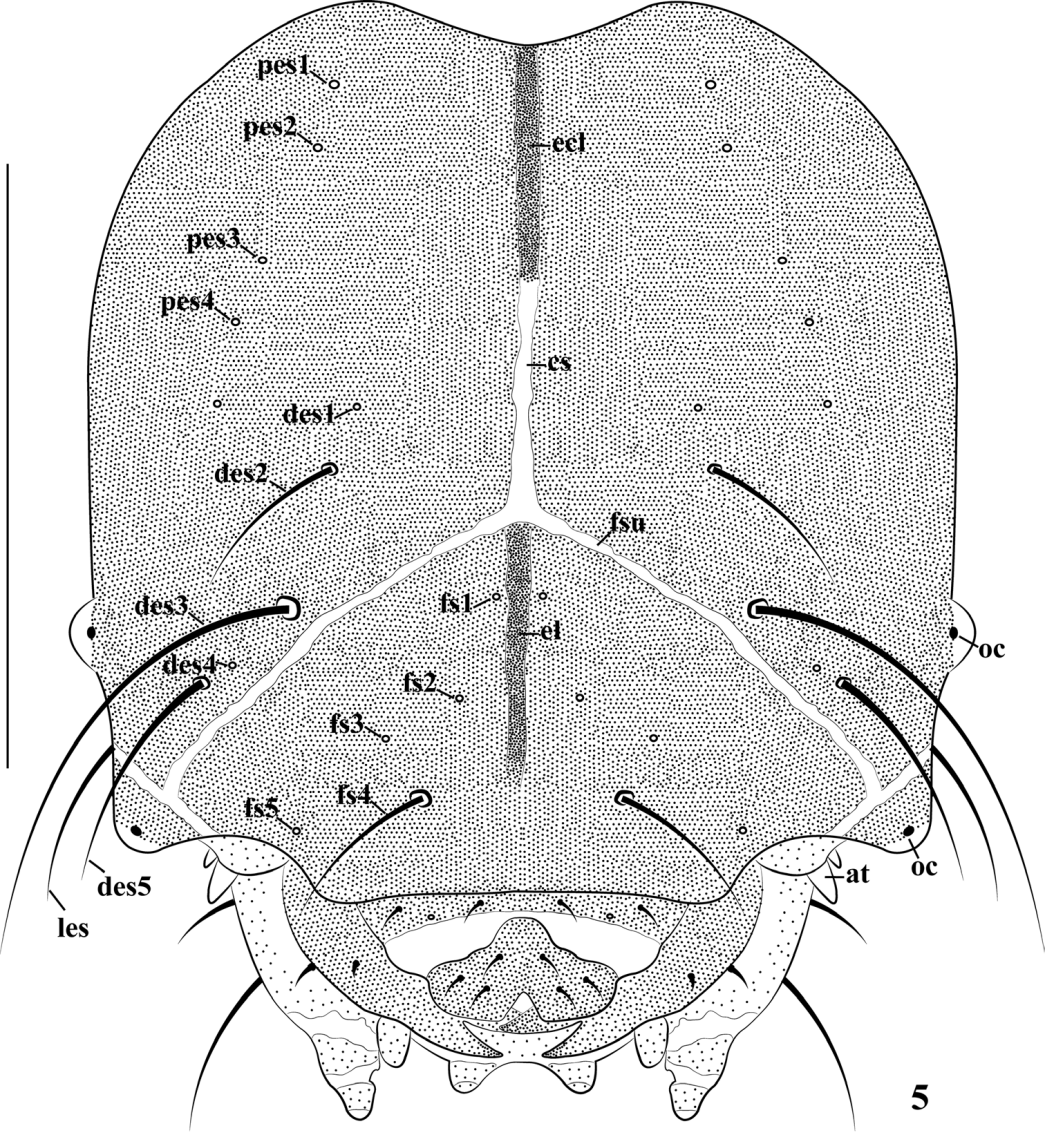
*Cionus olivieri*, mature larva, head, frontal view (at antenna, st stemmata, cs cranial suture, ecl ecdysial line, fsu frontal suture. Setae: *des* dorsal epicranial, *fs* frontal epicranial, *les* lateral epicranial, *pes* posterio-epicranial). Scale bar: 0.5 mm.

**Figures 6–11.**
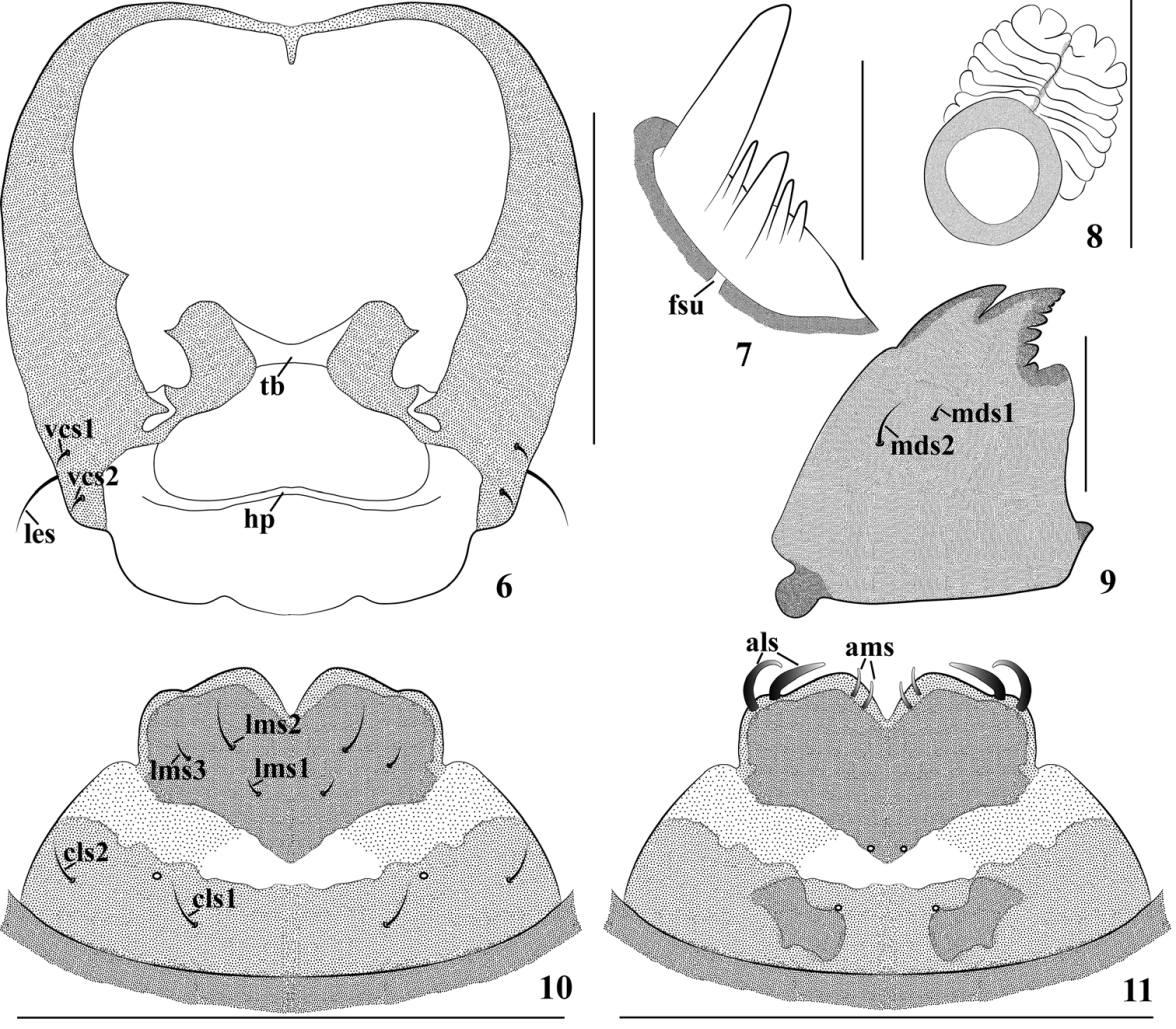
*Cionus olivieri* mature larva. **6** head, ventral view **7** antenna **8** spiracle **9** mandible **10** labrum and clypeus **11** epipharynx (*hp* hypopharyngeal bracon, *fsu* frontal suture, *tb* tentorial bridge). Seta(e): *als* anterolateral s., *ams* anteromedial s., *cls* clypeal s., *lms* labral s., *les* lateral epicranial s., *mds* mandible dorsal s., *ves* ventral epicranial s.). Scales bars: 0.5 mm (**6**), 0.25 mm (**10, 11**), 0.1 mm (**8, 9**), 0.025 mm (**7**).

*Head capsule* (Figs 5, 6). Head suboval and slightly rounded laterally, cranial suture undivided, wide, half-length of head. Frontal suture distinct, not extending to mandibular membrane. Endocarinal line present, reaching to half of frons. Anterior and posterior stemmata (st) present, projecting, anterior one located below stripe at side, externally close to antenna, posterior one located laterally. Setae on head piliform, varying in length, from very long to minute. Dorsal epicranium with three *des*; *des_1_* and *des_4_* reduced to a basal sensillum; *des_2_* approximately as long as one-third length of *des_3_*; very long *des_3_* located anteriorly on epicranium close to frontal suture; *des_5_* located anterolaterally, as long as two-thirds length of *des_3_*. Frons only with one *fs*; *fs_1_*, *fs_2_*, *fs_3,_* and *fs_5_* reduced to basal sensilla; long *fs_4_* located near epistoma. Epicranium with only one *les* as long as *des_1_*. Ventral epicranium with two, minute *ves.* Posterior epicranium with four, minute *pes_1_*–*_4_* and one sensillum. Postoccipital condyles distinct, hypopharyngeal bracon distinct. Tentorial bridge narrow, membranous in middle and half base of sides, strongly sclerotized at sides, with two pairs of acute auricular anterior projections.

*Antenna* (Fig. 7) located at the end of the frontal suture on each side, with one segment; sensory appendage (sensorium) three times as long as wide, circular in cross section, contiguous with frontal suture, with four conical sensillae.

*Clypeus* (Fig. 10) transverse-shaped, strongly sclerotized, with one sensillum and two *cls* of the same length, all in one line.

**Figure 12.**
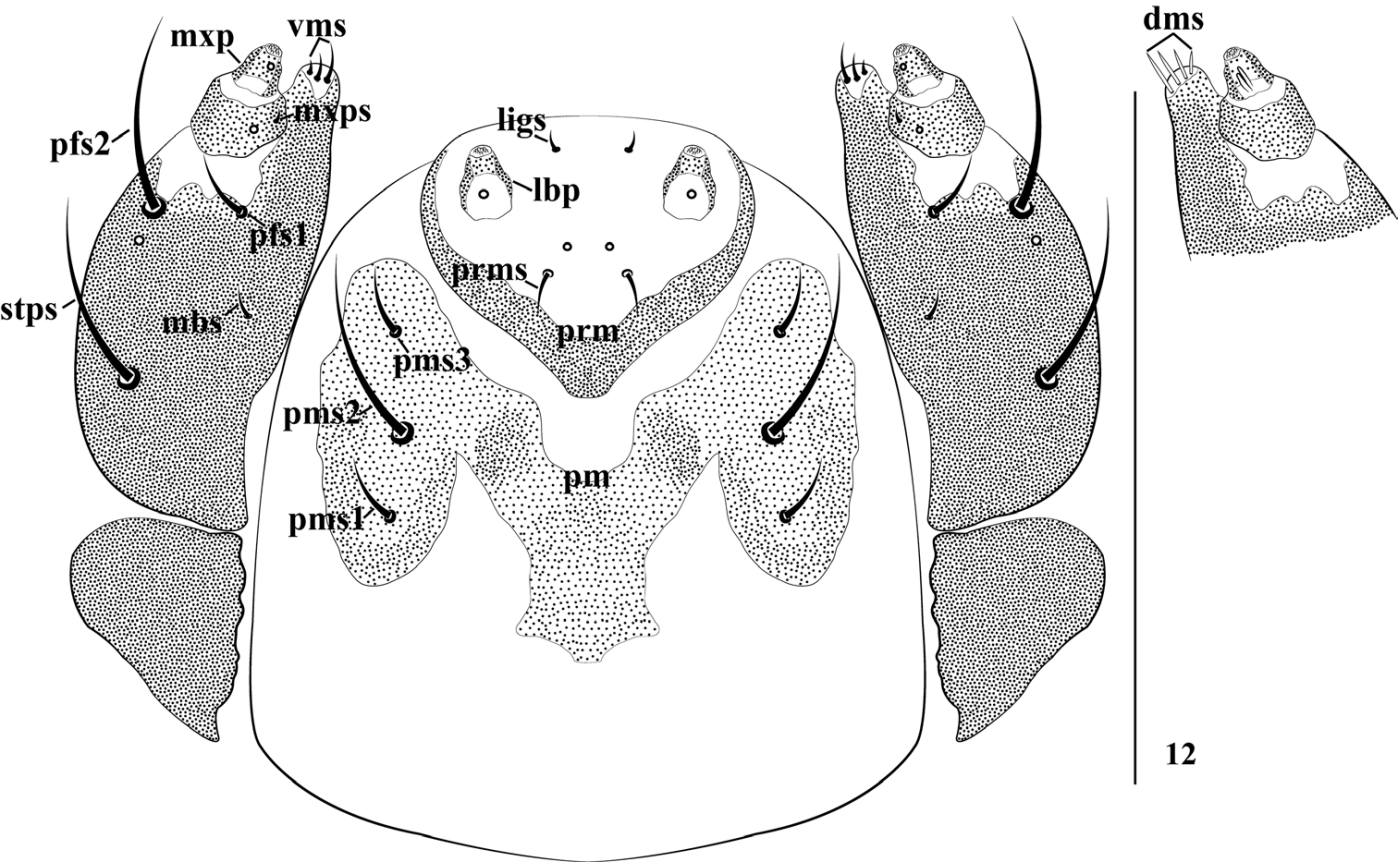
*Cionus olivieri* mature larva, maxillolabial complex, ventral view (lbp labial palpus, pm postmentum, prm prementum. Setae: *dms* dorsal malar s., *ligs* ligular s., *mbs* basoventral s., *mxps* maxillary palps s., *pfs* palpiferal s., *pms* postmental s., *prms* premental s., *stps* stipital s., *vms* ventral malar s.). Scale bar 0.25 mm.

*Mouthparts* (Figs 9–12). Labrum (Fig. 10) transverse-shaped, strongly sclerotized, deeply concave in the middle at apex, with three *lms*, very short *lms_1_* as long as *lms_3_*, and as long as half-length of short *lms_2_*, all localized centrally. Epipharynx (Fig. 11) with two small, stout, apically rounded *ams*; two relatively long, stout, apically rounded *als*; two epipharyngeal sensilla; *mes* and labral rods absent. Mandibles (Fig. 9) symmetrical, incisor section with two apical teeth and moderately rounded flange posterior to dorsal tooth, with many small teeth on inner side of anterior tooth, with one acute projection at base; molar section with two *mds*; *mds_2_* moderately long, *mds_1_* minute. Maxillae (Fig. 12) with maxillary palpi (mxp) two-segmented, basal segment with one tiny *mxps*, two clavate accessory appendages and one sensillum; distal segment sclerotized, with one sensillum, apex flattened with dense short irregular spiculate setae. Mala with four dorsal robust *dms*, gradually reducing in length; with three short, more acute *vms.* Stipes with one *stps*, two *pfs*, one *mbs* and one sensillum, very long *stps* located submedially on venter of base, *mbs* minute, long *pfs_1_* three times longer than *pfs_2_*. Labium (Fig. 12) membranous excepting the premental sclerite; labial palpi with one segment, longer than wide, slightly globular at base, apex of palpi flattened with short, dense, irregular, spiculate setae, and one sensillum. Prelabium (prm) with sclerite distinctly dilated posteriorly and laterally, without posterior extension, cup-like, with one sensillum and one short *prms*; ligula with one tiny *ligs*. Postlabium (*plb*) with M-shaped sclerotization, with three *plbs* on sclerotized area, all setae separated from each other by about the same distance; short *plbs_1_* and *plbs_3_* as long as one-third length of *plbs_2_*.

*Thorax* (Fig. 2). Prothorax with pronotal shield partly sclerotized on dark brown smooth plate; with nine *prns*: two short ones on sclerotized area, two long and two short ones placed anteriorly, and three placed more medially; bicameral spiracle intersegmental between pro- and mesothorax, air-tube subequal to diameter of circular peritreme; pleural lobe with two *ps*; pedal area without setae; eusternum with one *eus.* Mesonotum with two folds, prodorsum without seta, postdorsum with two transversally aligned *pds* of the same length; epipleurum with one long *eps*; pleurum with one long *ps*; setae of pedal area and eusternum identical to that of prothorax. Chaetotaxy of metathorax identical to that of mesothorax.

*Abdomen* (Figs 2–4). Abdominal segments I–VII of almost equal lengths, remaining abdominal segments gradually decreasing in width posteriad. Tergites on abdominal segments I–VII with three folds, prodorsum wide and flat, mesodorsum narrow, with soft protuberance, postdorsum with soft protuberance; tergite on abdominal segment VIII with two folds, and on abdominal segment IX with no folds. Abdominal segment X reduced to four anal lobes of unequal size. Anus located subterminally. Spiracles (Fig. 8) bicameral, the eight abdominal spiracles located more ventrally up to ventral side of epipleurum, close to the anterior margin of abdominal segments I–VIII, size similar, each with two annulated air-tubes, pointing posteriad, air-tube subequal to diameter of circular peritreme. Abdominal segments I–VIII with three *pds*, located in one line, long *pds_1_*, two times longer than *pds_2_*, *pds_2_* as long as *pds_3_*; with two *ss*, long *ss_1_*, short *ss_2_*, located in small indefinitely folds; with two *eps*, transversally aligned, long *eps_1_*, short *eps_2_* as long as one-third length of *eps_1_*; with one long *ps*; and with one *eus.* Abdominal segment IX with one short *ds*, one slight long *ps* and one, minute *sts.* Abdominal segment X without setae.

**Figures 13–20.**
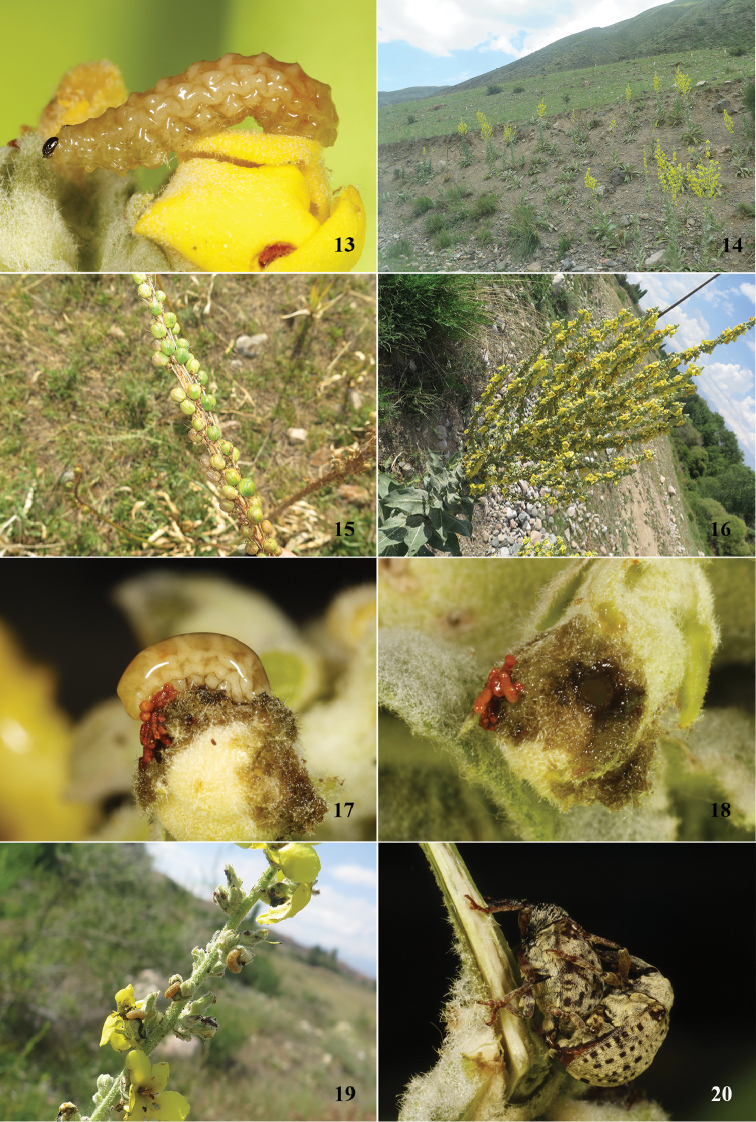
Biology of *Cionus olivieri* from Kyrgyzstan **13** larva on flower **14** habitat **15** seeds of host plant **16** flowering host plant **17** larva on feeding **18** feeding holes **19** damage on host plant by larva **20** copulating adults.

#### Biological notes

The biology of this species was studied on *Verbascum sinuatum* in southern France by Hoffmann (1958), where it has two generations, in June and August. The ectophagous larva digs a deep groove on the underside surface of a leaf. When mature, it builds a cocoon, where pupation takes place, on the same plant. The adults of the second generation hibernate in the soil. The same behavior was reported in other species of *Cionus* feeding either on *Verbascum* (Grandi 1929; Ruffo 1937; Hoffmann 1958) or *Scrophularia* (Read 1977; Räther 1989).

We collected larvae of *Cionus olivieri* (Fig. 13) from *Verbascum songaricum* Schrenk in early July in mountain slopes at 1546 m altitude (Fig. 14) in Kyrgyzstan. This plant, widely distributed in the temperate zone of Eurasia including the Caucasus, central Asia, and Tacheng, Xinjiang, China, was never previously reported as host of *Cionus*. Two-thirds of the host plants had already produced seeds (Fig. 15), while the remaining parts were still blooming (Fig. 16). Larvae were eating on the flower buds, with head, thorax, and part of the abdomen burrowed into the ovary and leaving red excrement, with a layer of clear mucus on the surface of the body (Fig. 17). The feeding holes were regular circles (Fig. 18). The most serious damage by the larvae reached 90% of one branch (Fig. 19), and most of the damaged buds could not bloom. We also observed many adults, more than 50% of which were mating (Fig. 20) and most of them were in the middle part of the host plants.

**Figures 21–22.**
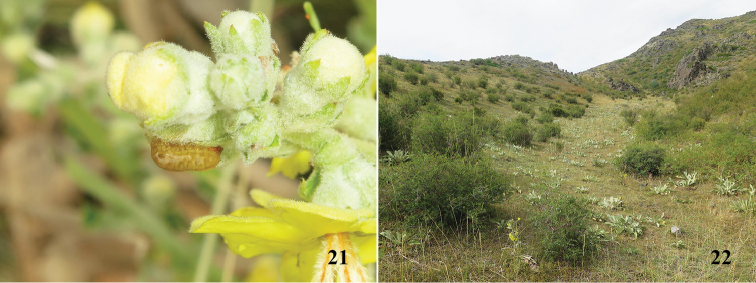
Biology of *Cionus olivieri* from Kazakhstan. **21** larva on bud **22** habitat.

Later, we collected larvae of *Cionus olivieri* (Fig. 21) from the same host plant species in mid-August in mountain slopes at 1577 m altitude (Fig. 22) in Kazakhstan. Damage of the larvae were similar to those in Kyrgyzstan. However, we did not find adults mating. Bearing in mind Hoffmann’s observations, we can assume that we probably collected the first generation in Kyrgyzstan and the second generation in Kazakhstan. However, in both situations, we did not find any cocoons on the plants (Fig. 15). This fact might be explained in two ways: both our observations were made before pupation or pupation happened in the soil. This second is unusual in *Cionus* but has been observed in *Cionus alauda* (Herbst, 1784) by Read (1977) and *Cleopus pulchellus* (Herbst, 1795) by Räther (1989).

## Discussion

### Comparison with larvae of other *Cionus* species

The larvae of eight *Cionus* taxa have been previously described (Grandi 1929, 1938; Scherf 1964; Lee and Morimoto 1988b). Unfortunately, comparing descriptions of some previously described *Cionus* species is problematic because of missing details on the chaetotaxy and/or the absence of quality drawings. Only the descriptions of four species were partly useful for comparison ‒ *Cionus helleri* Reitter, 1904 (Lee and Morimoto 1988b), *C. hortulanus* (Geoffroy, 1785) (Grandi 1929), *C. olens* (Fabricius, 1792), and *C. scrophulariae* (Linnaeus, 1758) (Grandi 1938) ‒ whereas the descriptions of another four ‒ *C. alauda* (Herbst, 1784), *C. tuberculosus* (Scopoli, 1763), *C. olivieri*, and *C. thapsus* (Fabricius, 1792) (Scherf 1964) – were almost completely useless because they were very lacking in details. In particular, the morphological description of *Cionus olivieri* larva by Scherf (1964) is scant, and there are only a few details, which can be easily compared with our detailed descriptions. There are only a few useful characters, e.g., body length, number of folds on thoracic and abdominal segments, but no valuable information about chaetotaxy (actually he presents two *des* and two *fs*, and not three *des* and one *fs*).

The mature larvae of the genus *Cionus* are probably characterized by three diagnostic features: the reduced number of setae (1) on the epicranium (only two or three *des* and one or two *fs*), and (2) on the epipharyngeal lining (only two *als*, two *ams*, and no *mes*), i.e., distinctly fewer than the most frequent number of setae in weevils, and (3) mandibles dentate or angulate internally near the base (for details, see Grandi 1929, 1938; Scherf 1964; Lee and Morimoto 1988a). The unique exception of the first two diagnostic features is the larva of *Cionus helleri*, which has the standard number of setae as in other weevils, i.e., five *des* and five *fs* (vs. two or three *des* and one or two *fs*); and eight epipharyngeal setae (vs. four epipharyngeal setae, two *als*, and two *ams*). This fact appears particularly interesting. Indeed, Košťál and Caldara (2019) recently noticed that *C. helleri* is unique in Palearctic *Cionus* in having three tubercles on the pronotum, and mucronate apices of the meso- and metatibiae in males as well as spines on the anterior tarsomere 1. The first two character states are particularly interesting from a taxonomic and phylogenetic point of view because tubercles on the pronotum are possessed only by several species of Afrotropical *Cionus* and some Oriental *Cleopus* (M. Košťál and R. Caldara, pers. obs.), whereas the presence of tibial unci in male is a plesiomorphic condition shared in other genera of Cionini, i.e., *Nanomicrophyes*, *Cleopus*, and *Stereonychidius* (Caldara and Korotyaev 2002). Therefore, in the light of the data on immatures, the systematic placement of *C. helleri* is presently problematic and interpretation and comparison of the distinctive larval characters is crucial and worthy of further study.

The count of setae on the epipharynx (especially *ams* and *mes*) in Curculionidae has not been completely resolved, but this has been discussed in previous papers (e.g., Tychiini: Skuhrovec et al. 2014, 2015; Gosik et al. 2017). In our case, the setae on the labrum and epipharynx can be easily confused due to the lack of resolution in most compound microscopes. Compared to the setae on the epipharynx of *Cionus helleri* (Lee and Morimoto 1988b) with eight epipharyngeal setae, there are only two very tiny *als*, two *ams*, and no *mes* in *C. olivieri* as listed also in other *Cionus* descriptions (Grandi 1929, 1938; Scherf 1964). We were not able to establish whether the difference is due to their being different species or they are erroneous observations. It is possible (C. Jiang and R. Zhang, pers. obs.) that the setae on the labrum and the edge part of epipharynx are more easily distinguishable by a scanning electron microscope.

The abdominal spiracles in Curculionidae are located mainly on the spiracular area, but we observed their position more ventrally up to the ventral side of the epipleurum in *Cionus olivieri*. A similar position of the spiracle in *Cionus* species is also shown on the drawings of Scherf (1964: figs 356, 366) and Lee and Morimoto (1988b: fig. 14B), but we have a different point of view on the status of setae around spiracles. Lee and Morimoto (1988b) reported them as *ss* setae, and setae above them as *pds_4_* and *pds_5_*. In our opinion, “our” *ss* setae are on a distinct lobe which is normally accepted as the spiracular area, and spiracles are placed more ventrally on epipleurum, known also for some Brachycerinae taxa (May 1993, 1994). The drawings by Scherf (1964: figs 356, 366) do not show any other setae above the abdominal spiracles.

### Comparison with larvae of related genera and tribes

With regard to the other genera of the tribe Cionini, only the immatures of three species were previously described, one belonging to *Cleopus* and two to *Stereonychus* (Scherf 1964; Lee and Morimoto 1988b). Unfortunately, as already emphasized for *Cionus*, two descriptions by Scherf (1964) for *Cleopus solani* (Fabricius, 1792) and *Stereonychus fraxini* (DeGeer, 1775) are problematic due to missing details on chaetotaxy, and it is almost impossible to compare it with *Cionus* species. Only a detailed larval description, that of *Stereonychus thoracicus* Faust, 1887 published by Lee and Morimoto (1988b), was useful. The mature larva of this species has similar chaetotaxy on the head (one *fs*, and no *mes*) as *Cionus* species, but there are some differences: *Stereonychus* has five *des* (in *Cionus* at most three), three *als* and three *ams* (in *Cionus* at most two *als* and two *ams*). It seems that mature larvae of *Cionus* (excluding *C. helleri*) + *Stereonychus* are characterized by six main diagnostic features: (1) labial palpi one-segmented, (2) labral rods absent, (3) pedal areas swollen to form large lobes or prolegs, (4) mandible with sharp apical teeth, (5) reduced number of *fs*, only one or two *fs*, on epicranium, and (6) reduced number of epipharyngeal setae (two or three *als* and two or three *ams*, but no *mes*), i.e., fewer than the most frequent number of setae recorded in weevils (for details, see Grandi 1929, 1938; Scherf 1964; Lee and Morimoto 1988a, 1988b). The first four diagnostic features in Cionini larvae are identical with *C. helleri* and also with Hyperini larvae (Lee and Morimoto 1988a, 1988b; Skuhrovec and Bogusch 2016), but this similarity with Hyperini is probably only due to the same ectophagous lifestyle.

Since it was hypothesized by a phylogenetic study based on morphological characters of imagos that the tribe Cionini might be the sister group of the tribe Mecinini (Caldara 2001) we also tried to compare the larvae of these two tribes. We found several distinct differential features: (1) bicameral spiracles in Cionini (vs. unicameral or bicameral spiracles in Mecinini); (2) dorsum of epicranium with two or three *des* (vs. five *des*); (3) frons with one or two *fs* (vs. three to five *fs*); (4) epipharynx without *mes* (vs. with one or two *mes*); and (5) mala with three to five *dms* and two to five *vms* (vs. six or seven *dms* and four or five *vms*). The count of palpomeres on the labial palpi was confirmed as one of the most important morphological characters of larvae in the tribe Mecinini (van Emden 1938; Skuhrovec et al. 2018; Gosik et al. 2020), but the larvae in the tribe Cionini have no variability in this character, and the labial palpi have only one palpomere.

We realize that a thorough study of the immatures of the genus *Cionus* and related genera and tribes is still impossible because of limited available knowledge. However, it is clear that a detailed description of immature stages is of primary importance for further studies on generic and intergeneric taxonomic relationships within Cionini and/or Curculioninae, as done in other groups of Curculioninae, such as Tychiini (Gosik et al. 2017) and Mecinini (Jiang and Zhang 2015; Skuhrovec et al. 2018; Gosik et al. 2020).

## Supplementary Material

XML Treatment for Cionus olivieri

## References

[B1] Alonso-Zarazaga M Lyal CHC (1999) A world catalogue of families and genera of Curculionoidea (Insecta: Coleoptera) (excepting Scolytidae and Platypodidae). Entomopraxis, Barcelona, 315 pp.

[B2] Alonso-Zarazaga M Barrios H Borovec R Bouchard P Caldara R Colonnelli E Gültekin L Hlaváč P Korotyaev B Lyal CHC Machado A Meregalli M Pierotti H Ren L Sánchez-Ruiz M Sforzi A Silfverberg H Skuhrovec J Trýzna M Velázquez de Castro AJ Yunakov NN (2017) Cooperative Catalogue of Palaearctic ColeopteraCurculionoidea. Monografías electrónicas SEA 8, Sociedad Entomológica Aragonesa SEA, 729 pp.

[B3] Caldara R (2001) Phylogenetic analysis and higher classification of the tribe Mecinini (Coleoptera: Curculionidae, Curculioninae). Koleopterologische Rundschau 71: 171–203.

[B4] Caldara R Korotyaev BV (2002) Taxonomic revision and reconstructed phylogeny of the weevil genus *Nanomicrophyes* Pic, 1908 (Coleoptera: Curculionidae, Curculioninae). Koleopterologische Rundschau 72: 183–195.

[B5] Emden F van (1938) On the taxonomy of Rhynchophora larvae (Coleoptera). Transactions of the Royal Entomological Society of London 87: 1–37. 10.1111/j.1365-2311.1938.tb01800.x

[B6] Gosik R Skuhrovec J Toševski I Caldara R (2017) Morphological evidence from immature stages further suggests Lignyodina being close to Tychiina (Coleoptera, Curculionidae, Curculioninae, Tychiini). Zootaxa 4320(3): 426–446. 10.11646/zootaxa.4320.3.2

[B7] Gosik R Skuhrovec J Caldara R Toševski I (2020) Immatures of Palearctic *Mecinus* species (Coleoptera, Curculionidae, Curculioninae): morphological characters diagnostic at genus and species level. ZooKeys 939: 87–165. 10.3897/zookeys.939.50612 32577083 PMC7297811

[B8] Grandi G (1929) Nota sul *Cionus hortulanus* Geoffr. Bollettino del Laboratorio di Entomologia di Bologna 2: 246–254.

[B9] Grandi G (1938) Morfologia ed etologia comparate di Insetti a regime specializzato. XV. La morfologia e l’etologia delle larve di tre Coleotteri delle famiglie dei Crisomelidi e dei Curculionidi. Bollettino del Laboratorio di Entomologia di Bologna 11: 1–16.

[B10] Hoffmann A (1958) Faune de France 62 Coléoptères curculionides (Troisième partie). Lechevalier, Paris, 1209–1839.

[B11] Jiang C Zhang R (2015) The genus *Gymnetron* from China with description of pre-imaginal stages of *G. miyoshii*, *G. auliense* and *G. vittipenne* (Coleoptera, Curculionidae). ZooKeys 534: 61–84. 10.3897/zookeys.534.5967 PMC4669937 26668548

[B12] Košťál M Caldara R (2019) Revision of Palaearctic species of the genus *Cionus* Clairville (Coleoptera: Curculionidae: Cionini). Zootaxa 4631(1): 1–144. 10.11646/zootaxa.4631.1.1 31712496

[B13] Lee CY Morimoto K (1988a) Larvae of the weevil family Curculionidae of Japan. Part 1. Key to Genera and the Short-nosed Group (Insecta, Coleoptera). Journal of the Faculty of Agriculture Kyushu University 33(1–2): 109–130.

[B14] Lee CY Morimoto K (1988b) Larvae of the weevil family Curculionidae of Japan. Part 2. Hyperinae to Cioninae (Insecta, Coleoptera). Journal of the Faculty of Agriculture Kyushu University 33(1–2): 131–152.

[B15] Marvaldi AF (1999) Larval morphology in Curculionidae (Insecta: Coleoptera). Morfologia larval en Curculionidae (Insecta: Coleoptera). Acta Zoologica Lilloana 45(1): 7–24.

[B16] May BM (1993) Larvae of Curculionoidea (Insecta: Coleoptera): a systematic overview. Fauna of New Zealand 28: 1–223.

[B17] May BM (1994) An introduction to the immature stages of Australian Curculionoidea. In: Zimmerman EC (Ed.) Australian weevils (Coleoptera: Curculionoidea).Vol. 2: Brentidae, Eurhynchidae, Apionidae and a chapter on immature stages. CSIRO, Canberra, 365–755.

[B18] Räther M (1989) Notes on four weevils in the tribe Cionini (Coleoptera: Curculionoide) associated with *Scrophularia nodosa* L. (Scrophulariae) Part I: Biology and ecology of the weevils. Bonner Zoologische Beiträge 40(2): 109–121.

[B19] Read RWJ (1977) Notes on the biology of *Cionus scrophulariae* (L.), together with preliminary observations on *C. tuberculosus* (Scopoli) and *C. alauda* (Herbst) (Col., Curculionidae). Entomologist´s Gazette 28: 183–203.

[B20] Rosenschoeld [= Rosenschöld] EM (1838) [new taxa]. In: Schoenherr CJ. Genera et species curculionidum, cum synonymia hujus familiae. Species novae aut hactenus minus cognitae, descriptionibus a Dom. Leonardo Gyllenhal, C. H. Boheman, et entomologis aliis illustratae. Tomus quartus. – Pars secunda. Parisiis: Roret, Lipsiae: Fleischer, 601–1121. [1122–1124 (Corrigenda)]

[B21] Ruffo S (1937) Osservazioni sull’etologia di un Curculionide a larva “pseudominatrice” (*Cionus olens* F.). Bollettino dell’Istituto reale di Entomologia dell’Università di Bologna 10: 167–177.

[B22] Scherf H (1964) Die Entwicklungsstadien der mitteleuropäischen Curculioniden (Morphologie, Bionomie, Ökologie). Abhandlungen der Senckenbergischen Naturforschenden Gesellschaft 506: 1–335.

[B23] Skuhrovec J Bogusch P (2016) The morphology of the immature stages of *Metadonus vuillefroyanus* (Capiomont, 1868) (Coleoptera, Curculionidae, Hyperini) and notes on its biology. ZooKeys 589: 123–142. 10.3897/zookeys.589.7847 PMC4926666 27408538

[B24] Skuhrovec J Gosik R Caldara R (2014) Immatures of Palaearctic species of the weevil genus *Tychius* (Coleoptera, Curculionidae): new descriptions and new bionomic data with an evaluation of their value in a phylogenetic reconstruction of the genus. Zootaxa 3839(1): 1–83. 10.11646/zootaxa.3839.1.1 25081903

[B25] Skuhrovec J Gosik R Caldara R Košťál M (2015) Immatures of Palaearctic species of the weevil genus *Sibinia* (Coleoptera, Curculionidae): new descriptions and new bionomic data with suggestions on their potential value in a phylogenetic reconstruction of the genus. Zootaxa 3955(2): 151–187. 10.11646/zootaxa.3955.2.1 25947846

[B26] Skuhrovec J Gosik R Caldara R Toševski I Łętowski J Szwaj E (2018) Importance of morphological characters of immatures for a better distinction of Palaearctic species of *Cleopomiarus* and *Miarus* from each other and other Mecinini (Coleoptera, Curculionidae, Curculioninae). ZooKeys 808: 23–92. 10.3897/zookeys.808.28172 PMC6305772 30598607

[B27] Smreczyński S (1976) Klucze do oznaczania owadów Polski. Czçsc XIX. Chrząszcze- Coleoptera. Zeszyt 98f. Ryjkowce-Curculionidae. Podrodzina-Curculioninae. Plemiona: Nanophyini, Mecinini, Cionini, Anoplini, Rhynchaenini. Polski Towarzystwo Entomologiczen, Warszawa, 115 pp.

